# Formulation of evidence-based messages to promote the use of physical activity to prevent and manage Alzheimer’s disease

**DOI:** 10.1186/s12889-017-4090-5

**Published:** 2017-02-17

**Authors:** Kathleen A. Martin Ginis, Jennifer Heisz, John C. Spence, Ilana B. Clark, Jordan Antflick, Chris I. Ardern, Christa Costas-Bradstreet, Mary Duggan, Audrey L. Hicks, Amy E. Latimer-Cheung, Laura Middleton, Kirk Nylen, Donald H. Paterson, Chelsea Pelletier, Michael A. Rotondi

**Affiliations:** 1 School of Health & Exercise Sciences, University of British Columbia, Kelowna, Canada; 2 Department Kinesiology, McMaster University, Hamilton, Canada; 3 Faculty of Physical Education and Recreation, University of Alberta, Edmonton, Canada; 4 Ontario Brain Institute, Toronto, Canada; 5 School of Kinesiology and Health Science, York University, Toronto, Canada; 6 ParticipACTION, Toronto, Canada; 7 Canadian Society for Exercise Physiology, Ottawa, Canada; 8 School of Kinesiology and Health Studies, Queen’s University, Kingston, Canada; 9 Department of Kinesiology, University of Waterloo, Waterloo, Canada; 10 Canadian Centre for Activity and Aging, Western University, London, Canada; 11 School of Health Sciences, University of Northern British Columbia, Prince George, Canada

**Keywords:** Exercise, Aging, Dementia, Fitness, Activities of daily living, Cognition, Health promotion, Messaging

## Abstract

**Background:**

The impending public health impact of Alzheimer’s disease is tremendous. Physical activity is a promising intervention for preventing and managing Alzheimer’s disease. However, there is a lack of evidence-based public health messaging to support this position. This paper describes the application of the Appraisal of Guidelines Research and Evaluation II (AGREE-II) principles to formulate an evidence-based message to promote physical activity for the purposes of preventing and managing Alzheimer’s disease.

**Methods:**

A messaging statement was developed using the AGREE-II instrument as guidance. Methods included (a) conducting a systematic review of reviews summarizing research on physical activity to prevent and manage Alzheimer’s disease, and (b) engaging stakeholders to deliberate the evidence and formulate the messaging statement.

**Results:**

The evidence base consisted of seven systematic reviews focused on Alzheimer’s disease prevention and 20 reviews focused on symptom management. Virtually all of the reviews of symptom management conflated patients with Alzheimer’s disease and patients with other dementias, and this limitation was reflected in the second part of the messaging statement. After deliberating the evidence base, an expert panel achieved consensus on the following statement: “*Regular participation in physical activity is associated with a reduced risk of developing Alzheimer’s disease. Among older adults with Alzheimer’s disease and other dementias, regular physical activity can improve performance of activities of daily living and mobility, and may improve general cognition and balance.*” The statement was rated favourably by a sample of older adults and physicians who treat Alzheimer’s disease patients in terms of its appropriateness, utility, and clarity.

**Conclusion:**

Public health and other organizations that promote physical activity, health and well-being to older adults are encouraged to use the evidence-based statement in their programs and resources. Researchers, clinicians, people with Alzheimer’s disease and caregivers are encouraged to adopt the messaging statement and the recommendations in the companion informational resource.

## Background

The current and impending public health impact of Alzheimer’s disease is staggering. Alzheimer’s disease is the most common form of dementia, characterized by progressive neural decline resulting in severe cognitive impairment, compromised physical ability, and loss of functional independence [1, 2]. The number of cases of Alzheimer’s worldwide is expected to increase from 30.8 million in 2010 to over 106 million in 2050. By 2050, it is projected that 1 in 85 adults worldwide will be living with the disease [3]. As no cure exists for Alzheimer’s disease, there is an urgent need for interventions to reduce the risk of developing it and to help manage the symptoms among those who have been diagnosed with it.

Physical activity may be a practical, economical, and accessible intervention for both prevention and management of Alzheimer’s disease. Engaging in routine physical activity could reduce the risk of developing the disease [4–11]. For individuals with Alzheimer’s disease, physical activity may help to mitigate and even improve some of the mental [4, 12–25] and physical [12, 19, 20, 22, 24, 26–30] symptoms. Moreover, a recent population-based analysis of seven potentially modifiable Alzheimer’s disease risk factors revealed that the largest proportion of disease cases in the United Kingdom, United States and Europe could be attributed to physical inactivity. A 10% reduction per decade in inactivity and the other risk factors was projected to reduce the prevalence of Alzheimer’s disease by up to 1.5 million cases in those countries [31]. These statistics provide a powerful case for the importance of public health campaigns and messaging to promote physical activity for the prevention and management of Alzheimer’s disease.

Evidence-based practice guidelines are an important tool to support the promotion of physical activity. Such guidelines stipulate the types, amounts, and intensities of physical activity needed for a particular population to derive certain benefits. For instance, the World Health Organization recommends that adults aged 18-64 should do at least 150 min of moderate-intensity aerobic activity throughout the week in order to achieve cardiorespiratory and muscular fitness, bone health, and to reduce the risk of non-communicable diseases and depression. Unfortunately, Alzheimer’s disease prevention and management are not included in this list of outcomes because the level of activity needed to achieve such benefits is not yet known [9]. Until appropriate dose–response data are available, it is impossible to formulate physical activity guidelines specifically for the prevention and management of Alzheimer’s disease (cf., [32]) or to endorse the current WHO guidelines as beneficial in this regard.

The absence of guidelines might imply that physical activity is not beneficial for those seeking to reduce their Alzheimer’s disease risk or to mitigate decline. Consequently, an important opportunity for public health promotion and disease prevention may be missed. Given the projected growth in Alzheimer’s disease cases over the coming decades [3], and the potential for physical activity to affect that trajectory [31], it is vital to communicate the Alzheimer’s disease-related benefits of activity to older adults.

Latimer-Cheung and colleagues have articulated the importance of using research evidence to formulate public health communications about physical activity [33]. Indeed, public health behaviour change programs are often criticized for lacking an evidence base [34], in part because health promoters traditionally operate in an environment that lacks systematic processes to consolidate research evidence into usable knowledge tools and resources. For instance, agencies responsible for promoting physical activity to Canadians with disabilities report that they want to use research evidence in their initiatives, but they often lack the resources to do so [35]. If health promoters do not have access to evidence-based messages and resources, then it is difficult for them to use evidence in their programs.

To address the evidence gap in public health physical activity messaging, Latimer-Cheung et al. published a case study in which the Appraisal of Guidelines, Research and Evaluation II (AGREE II) instrument was modified and applied to develop recommendations for constructing messages to support the Canadian Physical Activity Guidelines [32]. In general, the development process involved a literature review and the engagement of an expert panel to interpret the evidence and formulate recommendations based on the evidence. These steps were undertaken in a systematic manner that adhered to AGREE II standards for using evidence to develop and report clinical practice guidelines. Through this rigorous process, the authors demonstrated how to translate physical activity research into evidence-based messaging recommendations for use by groups with a vested interest in physical activity promotion.

Given the need for evidence-based messaging that communicates the public health benefits of physical activity for preventing or managing Alzheimer’s disease [36], coupled with the development of a systematic approach to formulating evidence-based physical activity messages [33], the purpose of the present project was to develop an evidence-based statement about the benefits of physical activity for preventing and managing Alzheimer’s disease.

### Background and project overview

The first author was contacted by a provincial, non-government organization that was interested in working with scientists to develop evidence-based messages and knowledge products to raise local (i.e., provincial) awareness regarding the benefits of physical activity for the prevention and management of Alzheimer’s disease. The first-author--a researcher with expertise in developing physical activity guidelines and evidence-based resources for adults with chronic disease and disability (KAMG)--and a scientist from the sponsoring organization (JA) agreed to co-direct the project. The project directors worked with an Appraisal of Guidelines Research and Evaluation II (AGREE-II) consultant and a researcher with expertise on exercise and Alzheimer’s disease (JH). Local stakeholders and scientists were involved in formulating the messaging statement and providing feedback.

The process for developing the messaging statement was guided by AGREE-II [37], an internationally recognized protocol for assessing the rigor, comprehensiveness and transparency of steps taken to formulate clinical practice guidelines. AGREE-II has been used previously as a framework for developing physical activity guidelines [38–40] and messages to support physical activity guidelines [33]. Paralleling the steps used by Latimer-Cheung et al., [31], the steps taken to develop the messaging statement were: a) determine the scope and purpose of the statement; b) conduct a systematic review of relevant literature; c) host a consensus meeting to formulate the statement; d) disseminate the statement for stakeholder feedback; (e) finalize the statement; and (e) review of the statement and this document by an AGREE II consultant. Each of these steps are described in the Methods section.

## Methods

### Statement scope and purpose

The following were determined by the project directors and confirmed appropriate by the expert panel members (see Table 1).

**Table 1:** Expert panel

Name	Expertise and Institution	Role(s)
Jordan Antflick (PhD)	Knowledge Synthesis, Knowledge Translation, Dissemination: *Ontario Brain Institute*	Knowledge Broker
Chris Ardern (PhD)	Guideline Development, Content (exercise, epidemiology): *York University*	Content Expert-Physical Activity Epidemiology
Christa Costas-Bradstreet	Dissemination: *ParticipACTION*	Stakeholder, Dissemination
Mary Duggan	Knowledge Synthesis, Guideline Development and Dissemination: *Canadian Society for Exercise Physiology*	Stakeholder, Dissemination
Jennifer Heisz (PhD)	Knowledge Synthesis, Content (Alzheimer’s disease, exercise, aging): *McMaster University*	Content Expert- Alzheimer’s disease, Aging, Exercise, Cognitive Neuroscience
Audrey Hicks (PhD)	Knowledge Synthesis, Guideline Development, Content (exercise, aging, practice): *McMaster University*	Content Expert-Physiology
Amy Latimer-Cheung (PhD)	Knowledge Synthesis, Guideline Development, Content (disability, behavior change), Knowledge Translation: *Queen’s University*	Content Expert-Exercise Behavior Change
Hans Messersmith	Knowledge Synthesis, AGREE, Guideline Development: *McMaster University*	Panel Chair, Process Advisor
Kathleen Martin Ginis (PhD)	Knowledge Synthesis, Guideline Development, Content (disability, behavior change), Knowledge Translation: *McMaster University*	Leadership, Project Direction
Laura Middleton (PhD)	Content (exercise, cognition, Alzheimer’s disease, dementia: *University of Waterloo*	Content Expert-Exercise, Cognitive Aging and Alzheimer’s disease
Kirk Nylen (PhD)	Knowledge Synthesis, Knowledge Translation, Dissemination: *Ontario Brain Institute*	Knowledge Broker
Don Paterson (PhD)	Content (exercise, aging): *Western University*	Content Expert-Physiology, Aging
Katherine Rankin (BA)	Dissemination: *Dementia Alliance, Alzheimer Societies of Brant, Haldimand Norfolk, Hamilton Halton*	Content Expert – Alzheimer’s disease Stakeholder, Dissemination
Michael Rotondi (PhD)	Evidence Synthesis, Meta-analysis models: *York University*	Content Expert-Biostatistics
John Spence (PhD)	Knowledge Synthesis, Guideline Development, Content (physical activity, behavior change): *University of Alberta*	Content Expert-Exercise Behavior Change

- *Overall statement objective*: To provide an evidence-based messaging statement for the use of physical activity (a) to prevent Alzheimer’s disease, and (b) to help manage symptoms and complications of Alzheimer’s disease.
- *Clinical questions addressed by the statement*: Can physical activity help to prevent Alzheimer’s disease in community-dwelling adults? Can physical activity be beneficial for managing symptoms and complications associated with Alzheimer’s disease (i.e., cognitive, affective, behavioural, sleep, physical, activities of daily living [ADL] and quality of life [QOL] outcomes)?
- *Target population*: Older adults who wish to prevent Alzheimer’s disease AND older adults with a diagnosis of Alzheimer’s disease.
- *Potential users of the statement*: a) older adults and their families, (b) primary caregivers of older adults with Alzheimer’s disease, c) health care providers including primary care physicians, physiotherapists, kinesiologists, attendant care providers, certified exercise physiologists, and occupational therapists, and d) local service organizations--such as the Canadian Society for Exercise Physiologists (CSEP) and the Alzheimer Society of Ontario –and public health and physical activity promotional agencies (e.g., ParticipACTION).

### Systematic review of systematic reviews

A systematic review of systematic reviews provided the evidence base for the messaging statement. Because several systematic reviews have already been published on Alzheimer’s disease, other dementias and physical activity [7, 8, 16, 19], a decision was made to review these articles rather than conduct yet another review. A review of reviews has the advantage of facilitating comparison and synthesis of findings across multiple reviews that may vary in scope and quality. Smith et al.’s [41] methodology was employed to guide the review protocol and is described next.

#### Scope of the review; literature search strategy and screening

The following inclusion criteria were set: English-language systematic reviews or meta-analyses examining the benefits of physical activity for either the management or prevention of Alzheimer’s disease in humans; reviews must have focused on *physical activity interventions* aimed at decreasing symptoms (e.g., declines in cognitive function, QOL, etc.) or managing Alzheimer’s disease; or *longitudinal/cross-sectional studies* that evaluated the role of physical activity in reducing the risk for Alzheimer’s disease. A research assistant developed the search strategy in consultation with the project directors. The search included PubMed and Cochrane Library databases (2003-August 2013) along with a hand search from reference lists of other papers.

To identify reviews of physical activity for *managing* Alzheimer’s disease, databases were searched for keywords: physical activity AND dementia AND reviews. This yielded 424 citations. An initial scan of these citations revealed that most reviews consisted of studies that included people with other dementias, not just Alzheimer’s disease. Though Alzheimer’s disease is the most common form of dementia, different pathologies can underlie dementia syndrome and most reviews did not distinguish participants based on their pathologies. Given the state of the literature, a decision was made to broaden our inclusion criteria to include reviews that focused on exercise to manage Alzheimer’s disease as well as other dementias.

The title and abstract of each citation were scanned and papers that were clearly outside the scope of the review were excluded; 20 reviews remained. The research assistant and one of the authors then reviewed the full text of these 20 articles and 14 met our inclusion criteria. To identify reviews of physical activity to *prevent* Alzheimer’s disease, a secondary search of the 424 citations was conducted using keywords: physical activity AND Alzheimer’s disease AND prevention AND reviews, yielding 60 citations. After scanning titles and abstracts, 19 reviews remained that focused specifically on prevention of Alzheimer’s disease (not the prevention of other dementias). After full text reviews, 6 of these 19 articles met our inclusion criteria. An updated literature search was completed in November 2015, and seven new reviews were added (one on prevention, six on management), resulting in a total of 20 reviews on management and seven reviews on prevention.

#### Data extraction and assessment of methodological quality

Individually, the research assistant and a study author extracted information from each review and assessed each review’s methodological quality using the 11-item *A Measurement Tool to Assess Systematic Reviews* (AMSTAR; http://www.amstar.ca/Amstar_Checklist.php) [42]. A score of 0–4 indicates low methodological quality, 5–8 indicates moderate methodological quality, and 9–11 indicates high methodological quality. The reviewers were not blinded during these steps. The extractions were completed in triplicate and AMSTAR evaluations were completed in duplicate. Any discrepancies were resolved through conversation until 100% agreement was achieved. Higher quality reviews were weighted more heavily than lower quality reviews when deliberating the evidence.

### Stakeholder involvement

Stakeholders representing various local interest groups (service providers, qualified exercise professionals), physical activity promoters, and knowledge brokers participated in the expert panel (Table 1) by developing and refining the messaging statement, and creating a supporting informational resource. Recognizing that some potential statement users were not on the panel, the statement was circulated to physicians who treat patients with Alzheimer’s disease and they provided anonymous feedback (*N* = 6). Healthy older adults drawn from an exercise and wellness program (*N* = 15) were given a paper copy of the statement and supporting resource and were directed to an online questionnaire to provide anonymous feedback (see Table 2). In addition, caregivers (*N* = 5) of older adults who participated in an exercise program for people with Alzheimer’s were given a paper copy of the statement and resource and completed a paper version of the questionnaire items shown in Table 2.

**Table 2:** Ratings of the statement and informational resource (i.e., “the toolkit”) obtained from health care providers and older adults

	Health care providers					
	n	M (SD)		Range of responses		
In your opinion, is the toolkit appropriate for all community-dwelling individuals with Alzheimer’s disease?	5	4.40 (.55)		4–5		
In your opinion, does the toolkit provide useful information for people with Alzheimer’s disease?	5	4.80 (.45)		4–5		
In your opinion, does the toolkit provide useful information for health care practitioners?	5	4.40 (.55)		4–5		
How confident are you that a client with Alzheimer’s disease could engage in enough physical activity each week to meet the current physical activity guidelines?	5	3.00 (.71)		2–4		
If given the opportunity, would you use this statement to recommend physical activity in your practice?	5	4.00 (.71)		3–5		
	Older adults			Caregivers		
	n	M (SD)	Range of responses	n	M (SD)	Range of responses
Does the statement provide useful information for older adults?	15	4.47 (.52)	4–5	5	4.20 (.45)	4–5
Does the statement provide useful information for families and caregivers of people with Alzheimer’s disease?	15	4.47 (.52)	4–5	5	4.20 (.45)	4–5
Is the statement clear regarding the benefits of physical activity?	15	4.40 (.63)	3–5	5	4.20 (.45)	4–5
In your opinion, is the toolkit appropriate for older adults with Alzheimer’s disease or those who want to prevent Alzheimer’s disease?	14	4.21 (.58)	3–5	5	4.20 (.45)	4–5
In your opinion, does the toolkit provide useful information for people with Alzheimer’s disease or those who want to prevent Alzheimer’s disease?	14	4.14 (.53)	3–5	5	3.80 (.87)	3–5
In your opinion, does the toolkit provide appropriate information to help older adults become more physically active?	14	4.21 (.43)	4–5	5	4.00 (.00)	4–4
In your opinion, does the toolkit provide clear information on the benefits of physical activity for preventing Alzheimer’s disease?	15	4.00 (.65)	3–5	5	4.00 (.00)	4–4
In your opinion, does the toolkit provide clear information on the benefits of physical activity for managing Alzheimer’s disease?	15	3.93 (.59)	3–5	5	4.20 (.45)	4–5

Note. All responses were made on a scale ranging from 1 to 5, with higher scores indicating more favourable ratings

### Consensus meeting

In September 2013, an expert consensus panel was convened for a 1-day meeting to review the evidence and formulate the statement. The meeting was chaired by one of the project directors and an AGREE II expert. Panel members included ten university-based researchers with expertise that spanned relevant content areas, knowledge synthesis and physical activity guideline development, along with five stakeholders representing health care professional groups and service organizations. The research assistant involved in the systematic review was also present. Given the importance of evaluating the research evidence with consideration of the context in which a resulting knowledge product will be disseminated [43], all but one panel member was based in the same province as the sponsoring organization and were thus familiar with the local context in which the knowledge products would be employed.

Prior to the meeting, all panel members received tabular summaries of the systematic review evidence (versions of Tables 3 and 4). The Chair began the meeting with an overview of AGREE-II and the process to be used to formulate the statement. Next, the chair presented the results from the systematic review of reviews on the use of physical activity to manage Alzheimer’s disease, followed by the systematic review of reviews on physical activity for prevention of Alzheimer’s disease. After each presentation, panel members discussed the strength, quality and quantity of evidence. Through these discussions, the panel came to unanimous agreement that insufficient quality evidence was available to produce a specific physical activity guideline (i.e., a prescription) for the prevention or management of Alzheimer’s. The panel agreed, however, that sufficient quality evidence existed to produce a consensus statement regarding the use of physical activity for these purposes.

**Table 3:** Summary of reviews examining the effects of exercise interventions on symptoms associated with Alzheimer’s disease and related dementias

Review	Quality score	# Studies in review^a^	Type	Characteristics			Outcomes		
				Participants	Design	Interventions	Physical	Psycho-logical	ADL and quality of life
Blankevoort et al., 2010^b^ [26]	9	16	NR/MA	Elderly (mean age >70 years) with dementia	10 RCT, 6 case series	Various structured exercise programs	*Physical Function:* ↑ Gait Speed, fast (k = 2) ES = 0.14; ↑ Gait speed, normal (k = 6) ES = 0.29; ↑ Endurance (k = 5) ES = 1.08; ↑ Lower extremity strength (k = 7) ES = 0.85 ↑ Functional mobility (k = 6) ES = 0.28 *Balance and Falls:* ↑ Balance (k = 5) ES = 1.76		↑ ADL (k = 4) d = 0.68
Boote et al., 2006 [27]	8	1	NR	Mod-severe AD	RCT	Group exercise	*Physical Function:* <>Functional ability (0/1), ↑ Physical Therapy Assessment (1/1) *Balance and Falls:* ↑ Balance (1/1)		
Brett et al., 2015 [12]	9	12	SR	Dementia living in nursing home	RCT	Any PA	*Physical Function:* ↑ Mobility (3/5) *Balance and Falls:* ↑ Balance (1/2)	*Cognition:* ↑ Cognition (5/7); *Affect:* ↑ Mood (3/4); ↓ Agitation (1/1)	↑ ADL (3/5)
Burton et al., 2015 [28]	11	4	SR/MA	Dementia living in the community	3 RCT and 1 quasi-experi-mental	Strength, balance and mobility exercises	*Balance and Falls:* ↓ Falls (k = 2) MD = -1.06*; <>Fall risk (k = 2) MD = -0.1; <>Balance (k = 2) MD = 0.51		
Cooper et al., 2012 [13]	10	1	NR	Dementia	RCT	Comprehensive exercise program			<>QOL (1/1)
de Souto Barreto et al., 2015 [14]	8	20	SR/MA	Dementia	RCT	Any exercise		*Affect:* ↓ Depression (k = 7) SMD= -0.31* *Behaviours:* <>Behaviours (k = 4) MD= -3.88	
Farina et al., 2014 [15]	10	3	MA	AD	RCT	Any exercise (min. 4 weeks)		*Cognition:* ↑ Global cognition (k = 3) SMD = 0.75*	
Forbes et al, 2013 [16]	11	16	CR	Older adults (>65 years old) with dementia	RCT	Any exercise		*Cognition:* ↑ Global cognition (k = 8) SMD = 0.55* *Behaviours:* <>Challenging behaviours (k = 1) SMD=-0.60 *Affect:* <> Depression (k = 5) SMD = -0.14	↑ ADL (k = 6) SMD = 0.68*
Forbes et al, 2015 [17]	11	17	CR	Older adults (>65 years old) with dementia	RCT	Any exercise		*Cognition:* <>Global cognition (k = 9) SMD = 0.43; [excluding moderate-severe dementia (k = 8) SMD = 0.21] *Affect:* <> Depression (k = 5) SMD = -0.14 *Behaviours:* <>Challenging behaviours (k = 1) MD= -0.60	↑ ADL (k = 6) SMD = 0.68*
Groot et al., 2016 [52]	9	18	MA	All dementia except those that affect motor system (e.g., Huntington’s, Parkinson’s)	RCT	Any physical activity		*Cognition:* ↑ Cognition (k = 16) SMD = 0.42*	↑ ADL (k = 4) SMD = 1.18*
Hermans et al., 2007 [18]	9	0	CR	Dementia living in domestic setting	RCT	Walking and exercise therapy		*Behaviours:* No studies of wandering met inclusion criteria	
Heyn et al., 2004 [19]	10	30	MA	Older adults (≥65 years) with cognitive impairment (MMSE <26)	RCT	Any exercise	*Physical Fitness:* ↑ Health-related physical fitness (k = 40) ES = 0.69*; ↑ Cardiovascular (k = 18) ES = 0.62*; ↑ Strength (k = 17) ES = 0.75*; ↑ Flexibility (k = 4) ES = 0.91* *Physical Function:* ↑ Functional performance (k = 20) ES = 0.59*	*Cognition:* ↑ Cognition (k = 12) ES = 0.57* *Behaviour:* ↑ Behaviour (k = 13) ES = 0.54*	
Jensen and Padilla, 2011 [29]	6	6	NR	Dementia	Mixed	Exercise and motor-based interventions for falls prevention (2 group-based; 4 individual)	*Balance and Falls:* ↓ Fall risk (3/4) ↑ Balance (1/1)		
Littbrand et al., 2011 [20]	9	10; results from 6 low quality studies not reported	NR	Dementia	RCT	Walking and combined exercise	*Physical Function:* ↑ Walking performance (2/2); <> Mobility (0/2) *Balance and Falls:* <> Balance (0/1)		↑ ADL (1/1)
O’Connor et al., 2009 [21]	8	1	NR	Dementia	RCT, RM	Any PA or exercise		*Affect:* ↑ Positive affect, (1/2); ↓ Negative affect, (1/2)	
Pitkala et al., 2013 [30]	8	20	NR	Dementia	RCT	Any PA	*Physical Function:* ↑ Physical function (16/20); ↑ Mobility or ↓ functional limitations (8/9 moderate-to-high quality studies)		
Rao et al., 2014 [22]	5	6	SR	Ambulatory older adults (>65 years) with AD	RCT with sample size >15	Aerobic, strength, and balanced or any combination of the three	*Physical Function:* Functional ability (k = 6) ES = 0.53*		↑ ADL (k = 6) ES = 0.80*
Thuné-Boyle et al., 2012 [23]	6	16	RCIA	Dementia	Exercise inter-vention studies (6) and reviews (10)	Any exercise		*Affect:* ↓ Agitation (4/4); ↓ Depression (4/8); *Behavior:* ↓ Wandering (1/2); ↑ Night time sleep (3/5)	
Yu, 2011 [24]	6	12	NR	AD	Experi-mental or quasi-experi-mental	Aerobic exercise (alone or combination; >2weeks)	*Physical Fitness:* ↑ 6 min walk (1/1); ↑ Strength (1/1); *Physical Performance* ↑ Physical performance (4/5)	*Cognition:* ↑ Global cognition, MMSE (4/4) *Affect:* ↑ Mood (4/6)	↓ ADL limitations (2/2)
Yu et al., 2006 [25]	8	18	NR	AD	Any	Aerobic exercise		*Cognition:* ↑ Global cognition (2/2)	

*Note: AD* Alzheimer’s disease, *ADL* activities of daily living, *CR* Cochrane review, *MA* meta analysis, *NR* narrative review, *PA* physical activity, *QOL* quality of life, *RCT* randomized controlled trial, *RCIA* rapid critical interpretive approach, *SMD* standard mean difference, *MD* mean difference, *ES* effect size; *k* number of studies

Values in parentheses indicate the number of studies or effect sizes in a review that addressed that outcome (denominator) and the number that indicated significant improvements (numerator)

*Significant effect size, *p* < .05

^a^For meta-analyses, ‘# of studies’ refers to the number of unique studies included in the reported meta-analyses

^b^Blankevoort et al. did not report the statistical significance of effect sizes nor did they report confidence intervals

**Table 4:** Summary of reviews examining whether physical activity in healthy older adults is associated with a reduced risk of developing Alzheimer’s disease and related dementias

Reference	Quality score	Type	Characteristics of included reviews				Conclusions
			# of studies^a^	Design	Participants	PA	
Beckett et al. 2015 [4]	7	MA	9	Prospective cohort studies	Cognitively healthy older adults, ≥65 years	Any PA	PA is associated with a ↓ risk of developing AD in adults 65 years and older. RR of .61, 95% CI 0.52-0.73 for physically active older adults compared to non-active counterparts.
Barnes et al., 2011 [5]	4	NR	2	Prospective cohort studies	No dementia diagnosis at baseline	Any PA	Of seven potentially modifiable risk factors examined, physical inactivity contributed to the largest proportion of AD cases in the US and a substantial proportion of cases globally.
Beydoun et al., 2014 [6]	7	MA	8	Cohort studies with sample size > 300	Generally healthy older adults	Any PA	RR of AD = 0.58 (0.49,0.70) for the group reporting the highest PA versus the lowest PA. PAR% = 31.9%, 95% CI 22.7–41.2%.
Daviglus et al., 2011 [7]	9	NR & MA	12	Cohort studies with sample size ≥ 300	General population in developed countries, ≥50 year	Self-reported PA.	NR: 8/12 studies reported a protective effect of moderate to high levels of PA on risk of AD; however, the associations were not always significant after adjusting for confounding factors or when looking across high and moderate activity levels. MA: Across 9 cohort studies, higher PA associated with ↓risk of incident AD (HR = 0.72); however, substantial heterogeneity among studies.
Hamer et al., 2009 [8]	11	MA	5	Prospective cohort studies	Diagnosis of dementia/AD	Any PA	PA ↓risk of AD by 45%. RR of AD = 0.55 for the group reporting the highest PA versus the lowest PA
Patterson et al., 2007 [10]	6	NR	3	Longitudinal cohort studies	Representative of Canadian demographic, exclusion of dementia at baseline	Any PA or energy expenditure	3/3 studies provided evidence that regular physical activity is associated with a reduced risk for AD.
Rolland et al., 2008 [11]	5	NR	24	Longitudinal epidemiological studies	No dementia diagnosis at baseline, ≥60 year	Any PA or energy expenditure	20/24 studies suggested a significant and independent preventive effect of physical activity on cognitive decline, or dementia, or AD risk. Physical activity could reduce the incidence of AD.

*Note.* ^a^For meta-analyses, ‘# of studies’ refers to the number of unique studies included in the reported meta-analyses

*AD* Alzheimer’s disease, *HR* hazard ration, *MA* meta-analysis, *NR* narrative review, *OR* odds ratio, *PA* physical activity, *PAR%* population attributable risk percent, *RR* relative risk

To assist in formulating the statement, as a starting point, panel members were presented with the following preliminary statements: “*Among people with Alzheimer’s Disease, physical activity can improve important aspects of well-being including physical fitness, physical performance, cognitive functioning and mood;”* and *“Habitual physical activity can reduce the risk of developing Alzheimer’s Disease*.” These statements were constructed by the lead author. The first statement was a summary of conclusions drawn in the reviews shown in Table 3, particularly those cited by Yu [24]. Yu’s conclusions were considered an appropriate starting point because they captured a broad range of outcomes. Note however, that Yu’s review did not consider the quality of the reviewed evidence so those conclusions could not be considered definitive. The second preliminary statement paralleled Hamer et al.’s [8] conclusion that “physical activity is inversely associated with risk of dementia”. In that meta-analysis, the quality of the evidence had been taken into consideration although the data were drawn from studies published in 2007 and earlier. The evidence was then discussed until the panel achieved a unanimous consensus statement.

Next, the panel discussed the potential health benefits and risks associated with the statement. The panel acknowledged the extensive body of evidence showing the wide range of health and fitness benefits that older adults can accrue from regular physical activity [9]. The panel also noted evidence that populations with dementia do not report considerable or consequential adverse events associated with physical activity [44].

The panel recommended that the evidence base be reviewed at least every three years to ascertain whether the messaging statement requires updating. During these reviews, consideration should be given to whether the quality and quantity of evidence have developed sufficiently to allow for formulation of physical activity guidelines. At this time, because only the initial messaging statement development process has been funded, the feasibility of ongoing updates is uncertain.

The consensus panel also discussed facilitators and barriers to implementing the messaging statement, including resource implications and informational needs. Panel members worked in sub-groups to identify content for an informational resource to support the uptake of the messaging statements. Discussions were guided by existing research on physical activity messaging and informational needs of older adults [45], along with consideration of dementia symptoms [46, 47]. The resultant recommended content could be generally categorized as clarification messages, motivational messages, and information for caregivers, and was subsequently given to a technical writer who drafted and wrote the content for the informational resource (http://www.braininstitute.ca/physical-activity-and-alzheimers-disease-toolkit).

Since the original consensus panel meeting, the panel has convened once by teleconference and twice by email to modify the statement based on the new evidence. An AGREE-II expert formally audited our procedures for developing the statement, using the AGREE-II Online Guideline Appraisal Tool (http://www.agreetrust.org/appraisal/15654) [37].

## Results

### Systematic review

With regard to *preventing* Alzheimer’s disease, physical activity was associated with a reduction in risk of Alzheimer’s disease in all seven review articles. There were 33 unique studies included in the reviews. These studies captured virtually any type of physical activity or energy expenditure (see Table 4). Two review articles were of high methodological quality [7, 8], four were of moderate quality [4, 6, 10, 11], and one was low quality [5]. Six of the seven reviews concluded that physical activity was associated with a significant reduction in risk of Alzheimer’s [4–8, 10], although one of the high quality reviews graded the quality of evidence as low [7]. The seventh review [11] noted that 20 out of 24 reviewed studies reported a significant association between physical activity and reduction of risk of Alzheimer’s disease, but the authors stopped short of making conclusions about the effects of physical activity because of an absence of RCT-derived evidence. The authors did, however, conclude that an active lifestyle seems to have a protective effect on brain functioning and may also slow the course of Alzheimer’s disease. Overall, the studies reported in the reviews provided consistent evidence that physical activity is associated with a reduced risk for developing Alzheimer’s disease.

With regard to *managing* Alzheimer’s disease and other dementias, there were 121 unique studies captured by the 20 systematic reviews. These studies included physical activity interventions involving structured exercise, group exercises, strength, balance and mobility exercises, walking and exercise therapy, and “any exercise” in general (see Table 3). Many studies had more than one outcome of interest --- the effects of physical activity on cognitive, affective, behavioural, physical (physical fitness, performance, balance), ADL and QOL were the outcomes examined in this review.

#### Cognition

Eight reviews reported on cognition and included from two [25] to 12 [19] studies. Six of the reviews were of high methodological quality, with five out of six providing evidence of positive effects of physical activity on cognition. Specifically, four reviews that included meta-analyses yielded significant average effect sizes, expressed as standardized mean group differences, ranging from 0.42 to 0.75. The fifth review found that exercise improved cognition in five of seven studies [12]. Whereas a 2013 Cochrane review found significant effects on cognition [16], the most recent (2015) Cochrane review [17], included just one additional trial [16] but found no significant effect (*p* = .08) and rated the available evidence as very low quality. The other two reviews were narrative reviews of moderate quality; both concluded exercise is a promising intervention for improving cognition [24, 25]. It is important to note that most of the studies included in the reviews employed a global measure of cognitive impairment, such as the Mini Mental State Examination [48] or the Montreal Cognitive Assessment [49], rather than measures of specific aspects of cognitive function. Taking this factor into consideration, overall, there is promising evidence that physical activity may have positive effects on global cognition. However, given the conflicting conclusions from the two recent Cochrane review [16, 17], no firm conclusion can be made.

#### Affect

Seven reviews examined affect-related outcomes and consisted of one to eight studies. Two Cochrane reviews of high methodological quality found no significant effect of physical activity on depression [16, 17]. One meta-analysis of moderate quality found that physical activity reduced depression [14]. Four other reviews, one of high quality [12] and three of moderate quality [21, 23, 24], all reported that some studies showed exercise can alleviate depression or enhance mood whereas other studies did not. Taken together, the extant research provides no consistent evidence that physical activity improves depression or other aspects of mood in this population.

#### Behaviours

Six reviews examined the effects of physical activity on challenging behaviours associated with dementia. Two of these reviews specifically addressed wandering; one was a high quality Cochrane review but the authors did not find any suitable studies to include in their review [16]. The other was of moderate quality and reported short-term decreases in wandering in one of two included studies [23]. The latter review also addressed nighttime sleep [23] and reported that three of the five included studies showed improvements. The other two reviews examined a range of challenging behaviours such as aggression, restlessness, wandering, and rummaging. Two were high quality Cochrane reviews [16, 17] that consisted of a single study and found no significant effects of exercise. The other two were moderate quality meta-analyses; one of which included 13 studies and found significant effects of physical training across a range of behavioural outcomes [19] and the other included seven studies which found no effect [14]. Based on these reviews, there is no consistent evidence that exercise improves challenging behaviours.

#### Physical outcomes

When reviewing the literature on physical outcomes, it became apparent little consistency existed across the reviews, and across the studies captured by those reviews, in operational definitions and measures of physical outcomes such as “physical fitness,” “mobility,” “physical function,” and “physical performance”. For instance, measures of walking performance were classified as an index of physical fitness in one review [24], physical function in another review [20], and reflected in ADL measures [50]. We have retained the original nomenclature of each review article to categorize the physical outcome measures; however, it is important to note that the categories are not clearly defined nor are they mutually exclusive.

#### Physical fitness

Two reviews examined outcomes that their authors categorized as “physical fitness” [19, 24]. One review was of high methodological quality [19] and included several meta-analyses of four to 40 studies that revealed significant effects of exercise training on cardiovascular, strength, flexibility, and overall fitness outcomes. A narrative review of moderate quality [24] reported on a single trial that improved 6-min walk distance and another that improved muscular strength.

#### Physical performance/function

Eight reviews reported on outcomes that their authors categorized as physical function or performance. Four were high quality. One high quality review reported average effect sizes (but did not report statistical significance or confidence intervals) ranging from 0.14 to 1.08 for the effects of physical activity on gait speed (fast and normal), endurance, lower extremity strength and functional mobility and concluded that multicomponent exercise training interventions can improve physical functioning [26]. Similarly, a high quality meta-analysis of twenty studies found significant medium-sized effects of exercise on measures of functional performance [19]. Of the two high quality reviews that consisted primarily of adults with Alzheimer’s disease living in residential care facilities, one reported significant improvements in two out of two reviewed studies of walking performance, but no improvements in mobility [20] whereas the other review reported improvements in mobility in three out of five reviewed studies [12].

There were four moderate quality reviews. One was a systematic review that reported that exercise increased functional ability [22]. The other three were narrative reviews [24, 27, 30]. Boote et al.’s [27] review included just one study, and focused on adults with moderate-severe Alzheimer’s disease. They concluded that regular exercise can significantly increase muscle strength and balance, but does not improve functional abilities as measured on the Changes in Advanced Dementia Scale [51]. In contrast, Pitkala et al.’s [30] review of 20 RCTs concluded there is consistent evidence that intensive exercise interventions enhance mobility and may also improve physical functioning if administered over the long-term. Likewise, Yu et al. [24] summarized the outcomes of five studies as showing improvement in physical performance among older adults with Alzheimer’s disease who participated in comprehensive exercise programs that had an aerobic exercise component.

Looking across the various physical outcomes, consistent evidence exists that physical activity can improve mobility--that is, people’s ability to walk, and to move around. Because so few studies employed true assessments of physical fitness (e.g., validated measures of cardiovascular endurance or muscle strength), no conclusions can be made regarding fitness outcomes.

#### Balance and falls prevention

Six reviews examined balance. One high quality meta-analysis [26] of five studies found a very large effect of physical activity on balance whereas another high quality meta-analysis of two studies found no effect [28]. The two other high quality reviews assessed adults in residential care. Littbrand et al.’s narrative review of a single study reported no effects on balance and Brett et al.’s systematic review of two studies reported effects on balance in only one. Two moderate quality narrative reviews, each including just one study, concluded that physical activity interventions improved balance [27, 29]. Two reviews directly examined falls; one of high quality and one of moderate quality, and both reported that exercise programs designed to prevent falls were found to be beneficial [29]. Taken together, there is promising evidence that physical activity may improve balance and reduce the risk of falls.

#### Activities of Daily Living (ADL)

Seven reviews addressed ADLs. Four high quality [16, 17, 26, 52] meta-analyses, two of which were Cochrane reviews, included four to six studies. All four reported medium to large-sized effects of physical activity on ADL. One of the Cochrane reviews concluded that there is promising evidence that exercise programs can significantly improve the ability to perform ADL [16]. Two high quality reviews that examined adults in residential care concluded that exercise improved or reduced the decline in ADL [12, 20]. Of the two moderate quality reviews, one reviewed six studies and reported a significant improvement in ADL [22] and the other reviewed [24] two studies and concluded that comprehensive exercise that includes aerobic exercise could help older adults with Alzheimer’s disease reduce ADL decline, and maintain basic and instrumental ADL. Taken together, the reviews provide consistent evidence that physical activity has positive effects on ADL.

#### Quality of Life (QOL)

One review examined QOL. This high quality narrative review, consisting of a single study, reported no effects of exercise on QOL [13]. At this time, there is insufficient evidence to draw any conclusions regarding the effects of physical activity on QOL in this population.

### The messaging statement

Drawing on discussions of the evidence presented in Tables 3 and 4, the panel achieved consensus on the following statement (see Table 5): “*Regular participation in physical activity is associated with a reduced risk of developing Alzheimer’s disease. Among older adults with Alzheimer’s disease and other dementias, regular physical activity can improve performance of activities of daily living and mobility, and may improve general cognition and balance*”. Panel members agreed there is insufficient or inadequate evidence to address whether physical activity can improve other outcomes such as affect, the risk of falling, quality of life, and challenging behaviours associated with dementia and Alzheimer’s disease.

**Table 5:** The messaging statement

“*Regular participation in physical activity is associated with a reduced risk of developing Alzheimer’s disease. Among older adults with Alzheimer’s disease and other dementias, regular physical activity can improve performance of activities of daily living and mobility, and may improve general cognition and balance.*”	

Although one review [20] indicated no major adverse effects of physical activity, few studies reported adverse effects [19, 20]. In generally healthy older adults, serious adverse events associated with physical activity are rare [53]. Regarding people with Alzheimer’s disease, the panel could not make an evidence-based decision regarding the risks associated with physical activity. Nevertheless, the panel acknowledged the low incidence of adverse physical activity-related events among people with dementia [44]. The panel agreed no evidence existed that physical activity is associated with increased risk of disease, or further progression or onset of Alzheimer’s disease.

### Stakeholder feedback

Stakeholder feedback was positive. For all three samples, mean ratings of appropriateness and utility of the physical activity messaging statement and informational resource ranged from 3.8 to 4.8 out of 5 (see Table 2). Older adults rated the clarity of information on the benefits of physical activity for preventing and managing Alzheimer’s disease at, or slightly below 4.0. This feedback resulted in minor wording changes to the informational resource.

### Editorial independence

The Ontario Brain Institute funded the messaging statement development project. Members of the Institute observed the consensus meeting but had no influence on the final statement whatsoever. No panel members declared a conflict of interest.

### AGREE-II evaluation

The messaging statement received an overall quality score of 6 out of 7 and was recommended for use. Table 6 shows ratings for each AGREE-II domain, areas identified for improvement, and subsequent modifications to this document that were made in response to the appraisal.

**Table 6:** AGREE-II domains, scores, areas for improvement, and responses/actions taken

Domain	Score	Areas for improvement in the report	Response/Action
1. Scope and Purpose	18/21	• Include specific outcomes of interest and setting to the clinical question • Additional details about the target population would have increased the rating (e.g., specific age ranges, specifying stage and/or severity of the disease)	• These details were added • These details cannot be provided given the limited research base
2. Stakeholder Involvement	19/21	None	
3. Rigour of Development	47/56	• Provide further details on the search for evidence (e.g., time periods searched, outcomes of interest, etc.) • Eligibility criteria for studies not explicitly stated/listed • Provide an explicit linking/identification of the key evidence underpinning the consensus statement	• Additional details have been added • An explicit statement has been added • Space restrictions preclude statement-by-statement links to the evidence; however it’s now noted that the evidence in Tables 3 and 4 has been used to guide the consensus statement
4. Clarity of Presentation	18/21	• The inclusion of a section or an appendix with the final consensus statement would make the statement more easily identifiable in the report	• A table/box was added to highlight the final statement
5. Applicability	21/28	• No explicit comments were included in the report concerning potential resource implications of applying the recommendations, nor was a formal assessment undertaken/reported	• Notes from the panel’s discussion of resource implications have been added
6. Editorial Independence	8/14	• An explicit statement regarding the funder was not included, nor was an explicit statement to indicate the views or interests of the funding body did not influence the final consensus statement	• An explicit statement has been added

## Discussion

A lack of evidence-based guidelines regarding the use of physical activity to prevent and manage Alzheimer’s disease may create a lost opportunity for promoting physical activity to older adults who may be motivated to be active for these reasons. To address this gap, our consensus panel formulated an evidence-based messaging statement by following the AGREE-II protocol. The first part of the messaging statement is wholly consistent with conclusions drawn in several reviews [4, 5, 8, 9] and speaks to the role of physical activity for preventing Alzheimer’s disease: “*Regular participation in physical activity is associated with a reduced risk of developing Alzheimer’s Disease*.” Though this statement is based exclusively on observational data, it satisfies several of Bradford Hill’s criteria for causation [54] including a strong and consistent association with temporal sequencing. It is also biologically plausible and consistent with emerging evidence that physical activity can change the structure and function of the brain. In particular, physical activity may mitigate age-related atrophy of the hippocampus, a key brain structure affected by Alzheimer’s disease that is critical for memory function [55]. The evidence reviewed did not differentiate between physical activities and sedentary tasks, however this distinction should be considered in future research given the emerging evidence that physical activity and sedentary behaviour may be independent predictors of health in aging [56, 57].

The second part of the statement reflects the best available evidence regarding the effects of physical activity on symptoms and complications associated with Alzheimer’s disease. The majority of reviews used the broader classification of dementia (rather than Alzheimer’s disease per se) as the study inclusion criterion and this qualifier is reflected in the statement: “*Among older adults with Alzheimer’s disease and other dementias, regular physical activity can improve performance of activities of daily living and mobility, and may improve general cognition and balance.*” This statement is generally consistent with the conclusions cited by several research groups [4, 20, 23, 29, 30]. However, some conclusions cited in those reviews were not carried over to our statement, including those pertaining to effects on affect, sleep, agitation, and wandering. These discrepancies are largely attributable to our consideration of review quality--higher quality reviews carried more weight in our deliberations--as well as the quantity and consistency of evidence across reviews. Parenthetically, given the inconsistencies across studies in the amount of exercise prescribed, and the disease severity of participants, it is perhaps not surprising that some areas of research have yielded inconsistent findings and that small changes to the evidence base can lead to new conclusions.

It is important to consider the implications of including other dementias in our analysis of the effects of PA on Alzheimer’s disease symptom management. Although Alzheimer’s neuropathology is present in up to 80% of dementia cases, each form of dementia is associated with a different symptom profile and rate of symptom progression [1]. Such heterogeneity means that the benefits of PA may differ by dementia subtype. That said, all forms of dementia impact the health and functioning of the brain and interfere with the individual’s ability to perform activities of daily living [1]. Moreover, commonly used pharmacological therapies are prescribed for symptoms that can be shared across Alzheimer’s disease and other dementias, even though these therapies may not be very effective and are commonly associated with adverse effects [58]. Therefore, the evidence-based messages regarding the benefits of regular PA for mitigating certain dementia symptoms with minimal adverse effects has important clinical relevance for individuals with Alzheimer’s disease and other forms of dementia. Future research is needed to evaluate whether the benefits of PA for dementia symptoms depend on symptom origin, profile or severity.

It is also important to consider the implications of the messaging statement and toolkit for Alzheimer’s disease patients living in residential facilities. Only one review focused exclusively on studies set in nursing homes with patients with mild to severe forms of dementia [12]. Although a limitation is that the 12 studies in that review had small samples, the authors of that review came to similar conclusions as the reviews involving community dwelling adults with Alzheimer’s. Thus, the messaging statement should be applicable to all individuals with Alzheimer’s disease regardless of their living arrangement. Of note, the review also concluded that interventions set in nursing homes had the greatest benefit when the PA program included a combination of aerobic, strength and stretching activities that were different from patients’ daily routine and were led by a trained physiotherapist. Programmers may find this information useful when implementing the messaging recommendations in nursing homes.

Though necessary for health education and promotion, guidelines and messaging statements are insufficient for motivating behaviour change in the absence of information on how to achieve the recommended behaviour [45]. Accordingly, the Ontario Brain Institute has produced *Boost Your Brain and Body Power - Physical Activity and Alzheimer’s Disease* [59]. This toolkit includes an informational resource that incorporates content generated by the expert panel, and promotes the Physical Activity Guidelines for Older Adults [32], while taking into account some of the unique concerns of a person with dementia (e.g., limited flexibility and balance, lapses in memory). The resource also describes the types of physical activities a person at risk for, or living with Alzheimer’s, should do and provides tips for staying safe and motivated.

### Applicability

We believe that the messaging statement and accompanying toolkit will have important implications for practice and research by providing inspiration for promoting physical activity for people with Alzheimer’s disease. One implication may be increased availability and development of fitness programs for people with Alzheimer’s disease. Furthermore, given a clear statement of the benefits of physical activity, service providers (e.g., exercise programmers, fitness centers, continuing care facilities) should be more inclined to offer programs tailored for older adults. The statement and accompanying informational resource should also reduce existing informational barriers that have discouraged health care practitioners from recommending physical activity. We also acknowledge potential resource implications of applying the statement. Individuals looking to increase their physical activity may incur financial costs associated with transportation, equipment or program fees. More staff and training may be necessary to facilitate increased demand and health care providers may require more time to discuss physical activity with patients during routine appointments.

Regarding research, the statement should stimulate more investigation of physical activity for preventing and managing Alzheimer’s disease, particularly research on the types and amounts of activity that yield benefit. Such research is needed in order to develop Alzheimer-specific physical activity guidelines, an important next step, given that clinicians were not particularly confident in Alzheimer’s patients’ ability to meet the general, national physical activity guidelines for older adults (see Table 2). As has been demonstrated through physical activity guideline development processes for other populations with chronic disease, mobility impairments and severe physical deconditioning [39, 40], lower volumes of exercise may be an appropriate recommendation for people with chronic conditions while still conferring significant benefits.

### Dissemination and implementation

The Ontario Brain Institute has released the messaging statement and informational resource on its website and in partnership with several local organizations including ParticipACTION, the Alzheimer Society of Ontario, and the Active Living Coalition of Older Adults. ParticipACTION has produced a webinar that supplements the statement. To reach the scientific community, these resources will be disseminated through academic journals and conferences. The OBI has also actively promoted (e.g., newsletters, webinars) the tool kit to clinicians and practitioners with the intent that it be shared with any newly diagnosed patients and their families.

### Surveillance

We are unaware of any efforts to monitor physical activity patterns of adults with Alzheimer’s disease. However, the Alzheimer’s Society of Canada has recently initiated exercise programs for older adults with Alzheimer’s disease and other dementias (cf. Minds in Motion). By tracking program participants--through accelerometry, or brief, validated questionnaires that can be completed by caregivers--it could be determined whether those who achieve Canada’s physical activity guidelines are deriving greater physical and health outcomes than those who do not. In addition, the Canadian Longitudinal Study of Aging [60] is tracking the activity patterns of healthy older adults and symptoms of Alzheimer’s disease/dementia over 20 years. These data will allow for ongoing surveillance of the association between physical activity and risk for Alzheimer’s disease.

### Limitations

We were unable to formulate a more specific exercise prescription for preventing or managing Alzheimer’s disease because the evidence is insufficient for determining dose–response relationships between physical activity and disease risk and outcome. The evidence is also sparse regarding the effects of physical activity on certain Alzheimer’s disease-related outcomes such as risk of falling, and QOL, which necessitated exclusion of these outcomes from our statement. We were unable to generate a statement for managing Alzheimer’s disease specifically because the majority of reviews used the broader classification of ‘dementia’ as the study inclusion criterion; this is an unfortunate characteristic of the extant literature. We also acknowledge that the expert panel and stakeholder surveys were comprised primarily of local (provincial) participants; this compilation ensured that international peer-reviewed research was used to formulate a locally relevant messaging statement and informational resource. And finally, we acknowledge that the literature search did not include grey literature (e.g., unpublished studies, organizational reports, materials not controlled by commercial publishers). However, given that the messaging recommendations are largely consistent with the conclusions generated in some of the most recent highest quality systematic reviews, we are confident that our search captured the most relevant research on physical activity and Alzheimer’s disease.

## Conclusions

Public health practitioners are often criticized for not incorporating research evidence in their behaviour change practices and initiatives [34]. Organizations that promote physical activity want to use research evidence in their practices, but are often limited in their capacity to do so [35]. In response to the needs of an organization that promotes brain health, this project has demonstrated how research evidence can be used to formulate evidence-based messages and knowledge products that can be disseminated by public health and other organizations to promote the use of physical activity to prevent and manage Alzheimer’s disease. Organizations that promote physical activity, health and well-being to older adults are encouraged to use the evidence-based messaging statement in their programs and resources. Researchers, clinicians, people with Alzheimer’s disease and caregivers are encouraged to adopt the messaging statement and the recommendations in the companion informational resource.

## Abbreviations

ADL: Activities of daily living
AGREE: Appraisal of Guidelines Research and Evaluation
AMSTAR: A Measurement Tool to Assess Systematic Reviews
QOL: Quality of life
RCT: Randomized Controlled Trial
WHO: World Health Organization
